# Rheumatoid arthritis as a risk factor for iron deficiency anemia: Results from a two-sample Mendelian randomization study

**DOI:** 10.1097/MD.0000000000045742

**Published:** 2025-10-31

**Authors:** Sijie Wang, Yue Lu, Chunlong Yang, Qiuhua Chen, Qingjun Pan

**Affiliations:** a Faculty of Chinese Medicine, Macau University of Science and Technology, Macao, China; b Clinical Research and Experimental Center, Affiliated Hospital of Guangdong Medical University, Zhanjiang, China; c Department of Anesthesiology, Affiliated Hospital of Guangdong Medical University, Zhanjiang, China; d Department of Rheumatology and Immunology, Affiliated Hospital of Guangdong Medical University, Zhanjiang, China.

**Keywords:** iron deficiency anemia, Mendelian randomization, rheumatoid arthritis

## Abstract

This study aims to investigate the potential causal relationship between rheumatoid arthritis (RA) and iron deficiency anemia (IDA) using a 2-sample Mendelian randomization approach. Additionally, we perform gene ontology (GO) and Kyoto encyclopedia of genes and genomes (KEGG) enrichment analyses to explore the biological significance of identified target genes. We utilized summary data from genome-wide association studies to conduct a 2-sample Mendelian randomization analysis. Genetic variants significantly associated with RA were employed as instrumental variables to assess the causal impact on the risk of IDA. GO and KEGG enrichment analyses were conducted using the R package cluster Profiler to understand the biological relevance of the single nucleotide polymorphisms target genes. Our findings indicate a significant causal relationship between RA and an elevated risk of IDA (inverse variance weighted, odds ratio = 1.05, 95% confidence interval = 1.012–1.09, *P* = .006). However, no significant association was observed between RA and anemia overall (inverse variance weighted, *P* = .342). GO enrichment analysis revealed that the most significant pathways in the biological process category were associated with interferon-gamma signaling. In the cellular component category, target genes were primarily linked to the endoplasmic reticulum and MHC protein complexes. Molecular function analysis showed involvement in binding and receptor activities related to the immune system. KEGG pathway analysis highlighted significant pathways such as Th1 and Th2 cell differentiation, Th17 cell differentiation, and inflammatory bowel disease, all closely related to immune response. This study provides evidence of a potential causal link between RA and IDA, suggesting RA as a risk factor for IDA in individuals of European ancestry. The enrichment analyses underscore the immune-related functional characteristics of the target genes, aligning with the autoimmune nature of RA. Further studies encompassing diverse populations are necessary to validate these findings and explore therapeutic targets.

## 1. Introduction

Rheumatoid arthritis (RA) is a systemic autoimmune condition marked by inflammation and joint degradation, stemming from abnormal immune and inflammatory responses. It has a prevalence ranging from 0.4% to 1.3%, and women are affected 2 to 4 times more frequently than men.^[1]^

Anemia, characterized by low serum iron concentrations despite sufficient iron stores, is commonly associated with RA and exemplifies anemia of chronic disease.^[2]^ Termed rheumatoid anemia, it stands as the most prevalent extraarticular manifestation of RA. Despite its prevalence in RA patients, the pathogenesis of rheumatoid anemia remains unclear.^[3,4]^ Involving factors such as shortened erythrocyte lifespan, inadequate bone marrow erythropoiesis, and abnormalities in iron metabolism.^[2,4–6]^

Iron deficiency anemia (IDA), a globally prevalent and treatable form of anemia, results from decreased hemoglobin synthesis leading to hypochromic and microcytic red blood cell production.^[7]^ While substantial progress has been made in understanding the molecular mechanisms of IDA in chronic diseases like RA, the association between RA and IDA remains unclear and debatable. Consequently, whether RA contributes to the risk of IDA remains uncertain. Clarifying this association holds potential to deepen our insights into the pathophysiology of IDA and autoimmune diseases, potentially paving the way for innovative therapeutic targets.

Chronic inflammation in RA elevates pro-inflammatory cytokines, notably interleukin-6, which stimulates hepatic production of hepcidin. As the central regulator of iron metabolism, hepcidin overexpression inhibits duodenal iron absorption and promotes iron sequestration within macrophages, culminating in functional iron deficiency and a heightened risk of IDA. Therefore, we hypothesize that genetic predisposition to RA may confer an elevated risk of IDA through this inflammation-mediated disruption of iron homeostasis, providing a strong a priori justification for our Mendelian randomization (MR) analysis.

MR, leveraging summary data from genome-wide association studies (GWAS), stands as a widely utilized approach to evaluate the relationships that could be causal between exposures and outcomes.^[8,9]^ In MR analyses, genetic variants significantly associated with the exposure serve as instrumental variables (IVs) to infer causality.^[10,11]^ If the exposure is causal, instrumental variables affecting the exposure will proportionally affect the results. In this study, we employed a 2-sample Mendelian randomization (TSMR) to scrutinize the potential causal relationship between RA and the risk of IDA.

## 2. Materials and methods

### 2.1. Data sources and single nucleotide polymorphism (SNP) selection

To explore the relationship between RA and anemia, we utilized 3 independent summary datasets for MR. The initial dataset for RA was retrieved from the IEU Open GWAS project (ID: “ebi-a-GCST90018910”), comprising 8255 cases and 409,001 controls of European ancestry. The second dataset, focusing on IDA, was sourced from the IEU Open GWAS project (ID: “ebi-a-GCST90018872”), including 12,317 cases and 468,624 controls of European ancestry. The third dataset, summarizing anemia data, was derived from the IEU Open GWAS project (ID: “ebi-a-GCST90038678”), involving 5259 cases and 479,339 controls of European descent.

To enhance the reliability and precision of causal inference between RA and anemia, we implemented several quality control steps for selection optimal instrumental variables. Initially, we identified SNPs significantly associated with RA and excluded those with a minor allele frequency below 0.01 to improved data quality. To maintain independence, we eliminated SNPs in linkage disequilibrium (*R*^2^ < 0.001, clumping distance = 10,000 kb) and excluded palindromic SNPs to ensure consistent effects.

Our selection criteria followed 3 key principles. Firstly, instrumental variables to be independent of confounding factors for both exposure and outcome. Secondly, selected variants had to show a significant association with the exposure, assessed through the *F* statistic (*F* value < 10 indicates weak association). Thirdly, instrumental variables were expected to influence outcomes solely through exposure, thus avoiding horizontal pleiotropy. These stringent criteria aimed to enhance reliability and validity in accordance with scientific reporting standards.

### 2.2. The assumptions of MR

**Figure 1.**
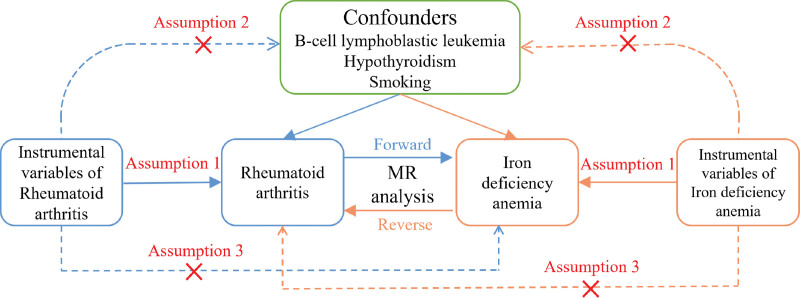
Schematic illustrating the MR analysis for the causal estimation of RA and IDA and anemia. The 3 key assumptions include: (1) the selected genetic variant as IVs should be significantly associated with the exposure, (2) IVs should not be associated with any confounding factors, and (3) IVs should affect the outcome solely through exposure without alternative pathways. IDA = iron deficiency anemia, IVs = instrumental variables, MR = Mendelian randomization, RA = rheumatoid arthritis.

A valid MR analysis must satisfy 3 critical assumptions: First, the genetic variants used as IVs must be robustly associated with the exposure (RA); Second, the IVs must not be associated with any confounders of the exposure–outcome relationship; and Third, the IVs must influence the outcome (IDA/anemia) exclusively through the exposure, with no alternative biological pathways (i.e., no horizontal pleiotropy). The analytical framework of this study, grounded in these assumptions, is illustrated in Figure 1.

### 2.3. MR estimates

In this investigation, we employed multiple MR methods to robustly estimate the causal effect of RA on IDA and general anemia. The primary analysis was conducted using the inverse variance weighted (IVW) method, which provides a consistent causal estimate under the assumption that all genetic variants are valid instruments (i.e., no horizontal pleiotropy).^[12]^

To assess the robustness of the IVW estimates and to evaluate potential violations of the MR assumptions, we performed a series of sensitivity analyses. First, we utilized the MR-Egger regression method, which can provide a valid causal estimate even when all genetic instruments are invalid, provided that the Instrument Strength Independent of Direct Effect assumption holds. The intercept term from MR-Egger regression serves as a test for directional horizontal pleiotropy. Second, we applied the weighted median estimator, which can provide consistent causal estimates even if up to 50% of the weight in the analysis comes from invalid instrumental variables.^[13]^ Third, we employed the weighted mode method, which remains valid when the largest plurality of instruments are valid, even if the majority are invalid.^[14]^ These methods serve as complementary sensitivity analyses to test the robustness of our primary IVW results.

To conduct a more comprehensive assessment of pleiotropy, we implemented the Mendelian Randomization Pleiotropy RESidual Sum and Outlier (MR-PRESSO) test.^[15]^ The MR-PRESSO method performs a global test for the presence of horizontal pleiotropy. Crucially, it then identifies and removes individual Our bidirectional TSMR analysis indicated a positive causal link between RA and IDA that are likely to be driving the pleiotropic signal, and provides a corrected causal estimate based on the remaining variants. This approach is distinct from, and complementary to, the MR-Egger method. While MR-Egger regression tests for and corrects a consistent directional pleiotropic bias across all instruments (via its intercept term), MR-PRESSO specifically targets and eliminates the influence of strong, individual pleiotropic outliers. Heterogeneity among the causal estimates of individual SNPs was quantified using Cochran *Q* statistic. To ensure that the overall causal estimate was not driven by a single influential variant, we performed a leave-one-out sensitivity analysis by iteratively removing each SNP and recalculating the IVW estimate. All analyses were conducted using the TSMR^[11]^ and MR-PRESSO^[15]^ packages within the R statistical software environment.

### 2.4. Enrichment analysis

To investigate the functional characteristics and biological relevance of the identified SNPs target genes, gene ontology (GO) and Kyoto encyclopedia of genes and genomes (KEGG) enrichment analyses were performed using the R package clusterProfiler.^[16]^ GO includes 3 categories: biological process, molecular function, and cellular component. KEGG pathway analysis provides insights into metabolic pathways.

## 3. Results

### 3.1. Instrumental variables selection

We selected SNPs strongly correlated with the exposure variable (*P* < 5 × 10^‐8^) and demonstrating high statistical power (*F* value > 10). Adhering to the criteria for MR analyses, we meticulously chose 24 SNPs associated with RA at a genome-wide significance level. Importantly, these selected SNPs were independent of each other in terms of linkage disequilibrium. All instrumental variables chosen exhibited *F*-values exceeding 10, indicating that the associated bias did not significantly impact the assessment of causal effects. This rigorous selection process ensures the robustness and reliability of our instrumental variables in investigating the potential causal relationship between RA and the outcomes under scrutiny.

### 3.2. Effect of RA on IDA and anemia

**Figure 2.**
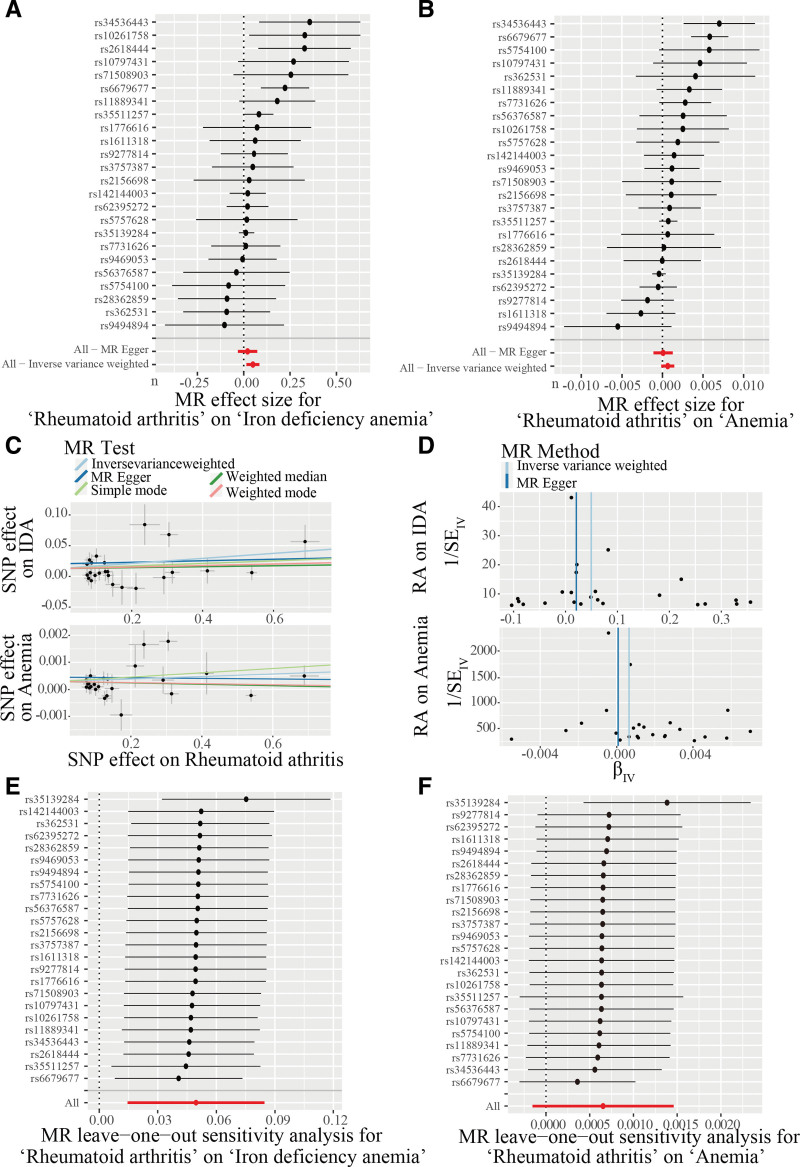
Combined forest plot of MR sensitivity analysis. MR-Egger and IVM methods indicate positive MR effect sizes (>0), suggesting a causal relationship between RA and both IDA (A) and anemia (B). Scatter plot illustrating the MR test results using 5 methods. The *X*-axis represents the SNP effect on RA, while the *Y*-axis represents the SNP effect on both IDA and anemia (C). Funnel plots depicting the MR results using IVW and MR-Egger methods for RA on IDA and anemia (D). Forest plots illustrating the results of the MR leave-one-out sensitivity analysis for RA on IDA (E) and anemia (F). IDA = iron deficiency anemia, IVW = inverse variance weighted, MR = Mendelian randomization, RA = rheumatoid arthritis, SNP = single nucleotide polymorphism.

The effects of RA on IDA and anemia are summarized in Figure 2A–C. Notably, the causal relationship between RA and IDA exhibited variations across the 5 MR methods. Results from the IVW method indicated a significant association between RA and IDA (IVW, odds ratio [OR] = 1.05, 95% confidence interval = 1.012–1.09, *P* = .006). We observed moderate heterogeneity in the causal estimates across the individual instrumental variables for this analysis (Cochran *Q P*-value = 0.064), which prompted the use of a random-effects IVW model to provide a more conservative and robust estimate. Conversely, the IVW method suggested no causal relationship between RA and anemia (IVW: *P* = .113) (Table 1). For this analysis, we noted significant heterogeneity (Cochran *Q P*-value < 0.001), likely reflecting the diverse etiologies encompassed within the broad phenotype of “anemia.” This heterogeneity was accounted for by using the random-effects IVW model. MR-Egger analysis provided evidence for the absence of underlying horizontal pleiotropy in both the relationship between RA and IDA (*P* = .169) and the relationship between RA and anemia (*P* = .217) (Table 1). The Cochran *Q* test results in Table 1 reveal no significant heterogeneity between RA and IDA, while heterogeneity is observed between RA and anemia (Fig. 2D). The “leave-one-out analysis” was conducted to illustrate the overall impact of SNPs on RA versus IDA and the effect of RA on anemia (Fig. 2E, F).

**Table 1:** Mendelian randomization estimates for the effect of rheumatoid arthritis on iron deficiency anemia and anemia.

Exposure	Outcome	No. SNPs	MR methods	*P*	OR	95% CI	Cochran *Q P*-value	Egger intercept *P*-value
RA	IDA	24	MR-Egger	.45	1.02	0.97–1.08	–	.17
			WM	.43	1.02	0.98–1.06	–	–
			IVW	.006	1.05	1.02–1.09	.06	–
			Simple mode	.428	1.03	0.96–1.11	–	–
			Weighted mode	.281	1.02	0.98–1.06	–	–
RA	Anemia	24	MR-Egger	.90	1.00	0.99–1.00	–	.22
			WM	.87	1.00	0.99–1.00	–	–
			IVW	.11	1.00	0.99–1.00	<.001	–
			Simple mode	.33	1.00	0.99–1.00	–	–
			Weighted mode	.99	1.00	0.99–1.00	–	–

The Cochran *Q P*-value and MR-Egger intercept *P*-value are primarily reported for the IVW and MR-Egger methods, respectively. The random-effects IVW model was applied in all analyses due to the presence of heterogeneity. *P*-values for the Egger intercept test the null hypothesis of no directional horizontal pleiotropy.

CI = confidence interval, IDA = iron deficiency anemia, IVW = inverse variance weighted, MR = Mendelian randomization, OR = odds ratio, RA = rheumatoid arthritis, SNP = single nucleotide polymorphism, WM = weighted median.

### 3.3. Effect of IDA and anemia on RA

We performed reverse MR analyses to examine the potential causal effects of IDA and anemia on RA. The analysis for IDA (comprising only 2 instrumental variables) was severely underpowered and showed no significant association with RA (IVW: *P* = .251). Similarly, the analysis for anemia on RA also yielded a null result (IVW: *P* = .180). Given the limited number of instruments, particularly for IDA, these reverse analyses contribute little to causal inference and their null findings must be interpreted with caution.

### 3.4. Enrichment analysis

**Figure 3.**
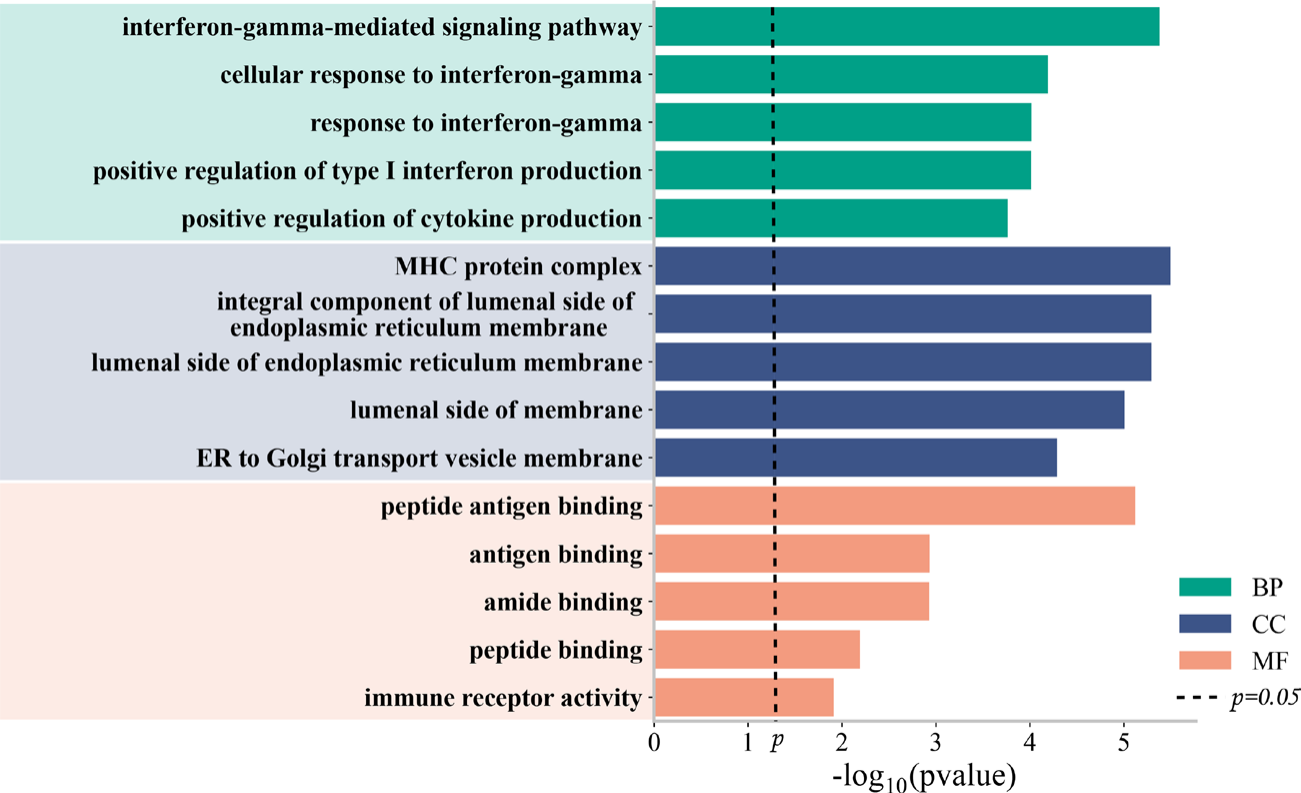
GO enrichment results for 3 terms. GO = gene ontology.

**Figure 4.**
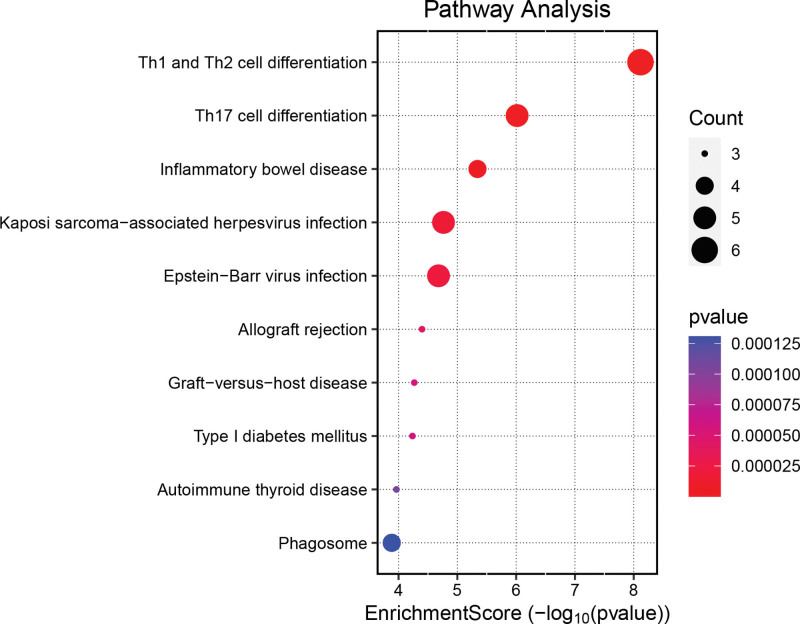
KEGG enrichment results. KEGG, Kyoto encyclopedia of genes and genomes.

GO enrichment analysis is commonly used to demonstrate interactions between genes and terms, while KEGG enrichment analysis illustrates the relationships between genes and functional pathways.^[17]^ As shown in Figure 3, the most significant pathways in the biological process category are all associated with interferon-gamma, including the interferon-gamma-mediated signaling pathway, cellular response to interferon-gamma, and response to interferon-gamma. In the cellular component category, target genes primarily focus on components related to the endoplasmic reticulum and MHC protein complexes. These processes are crucial for protein synthesis, modification, and transport, particularly in the processing and presentation of MHC molecules within the immune system, aligning with previous studies.^[18]^ Furthermore, in terms of molecular function, these genes are involved in binding and receptor activities related to the immune system, such as antigen binding, amide binding, peptide binding, and immune receptor activity. As shown in Figure 4, KEGG enrichment analysis identified the top 3 pathways as Th1 and Th2 cell differentiation, Th17 cell differentiation, and inflammatory bowel disease. inflammatory bowel disease, being an autoimmune disease, and the other pathways are all closely linked to the immune response.

## 4. Discussion

While the IVW method, considered the primary approach in MR analysis, yielded a statistically significant estimate for the effect of rheumatoid arthritis on IDA (OR = 1.05, *P* = .006), the lack of consensus across sensitivity analyses substantially undermines the robustness of this finding. The results from MR-Egger, weighted median, simple mode, and weighted mode methods were all nonsignificant and yielded effect estimates closer to the null. This notable inconsistency urges extreme caution in interpreting the IVW result and suggests that the observed nominal association may not be robust or reliably causal. Consequently, the primary IVW estimate should be viewed as, at best, indicative of a very weak and putative causal link that requires further validation.

In the current investigation, we conducted a bidirectional assessment of the causal relationship between RA and IDA and anemia. Employing a TSMR approach with GWAS summary statistics, our results indicated a potential causal link between RA and IDA, with an OR of 1.05 (95% confidence interval: 1.02–1.09). It is critical to emphasize that this effect size is very small. However, no significant association was found between RA and anemia. In the reverse TSMR analysis, there was no evidence of a causal relationship. Taken together, these findings suggest that the known clinical association may not be fully explained by shared genetics, and that RA may increase the risk of IDA primarily through nongenetic factors.

Notably, previous studies have explored the associations between RA and IDA, revealing that IDA is a prevalent issue in RA patients, with a reported prevalence of 60% according to specific research.^[19]^

A comprehensive literature review revealed that anemia is a prevalent comorbidity among individuals with RA. Estimates of mild anemia prevalence in this population range from 33% to 60%, and for children with juvenile rheumatoid arthritis, the prevalence of anemia varies from 35.8% to 52.3%.^[2]^ Notably, even in well-controlled cases, the frequency of anemia among individuals with RA is consistently around double that observed in the general population.^[20]^ However, recent findings from a study suggest that the occurrence of anemia in individuals with RA has significantly decreased, potentially attributed to earlier RA diagnosis and more effective disease control through modern therapeutic strategies.^[2]^ Our study, leveraging the extensive GWAS dataset available, specifically targeted individuals of European ancestry to minimize biases stemming from small sample sizes or ethnic variations. The findings of this study emphasize a significant link between RA and an increased risk of IDA. However, no significant association was found between RA and general anemia. Importantly, our analysis revealed no evidence of reverse causality between RA and IDA, and similarly, no evidence of reverse causality between RA and anemia was observed. The stark contrast between the high clinical prevalence of IDA in RA patients and the modest genetic association (OR = 1.05) identified in our study suggests that the observed clinical risk is predominantly driven by nongenetic factors (e.g., disease activity, treatments) rather than a strong shared genetic basis. The small magnitude of the genetic effect indicates that RA confers only a minor increase in the relative risk of IDA at the population level.

The mechanisms underlying these associations remain poorly understood, but some potential connections can be elucidated. The use of nonsteroidal anti-inflammatory drugs (NSAIDs) can potentially contribute to anemia in the body.^[21]^ NSAIDs function by inhibiting prostaglandin synthesis, crucial for iron absorption in the intestines. This inhibition, coupled with NSAID-related gastrointestinal toxicities like bleeding, may result in IDA.^[22,23]^ Prolonged NSAID use is also linked to decreased hemoglobin levels. Continuous, mild blood loss due to NSAID enteropathy could further lead to iron deficiency and anemia in long-term users.^[23,24]^ Monitoring hemoglobin levels is crucial for individuals employing NSAIDs over extended periods.

Moreover, chronic inflammation in RA can induce anemia of chronic disease, particularly in those with high disease activity. This type of anemia is characterized by disruptions in iron metabolism, leading to iron sequestration and functional iron deficiency.^[21,25]^

In this study, enrichment analysis was performed to understand the biological significance of the target genes. Gene enrichment analysis reveals the functional characteristics and biological relevance of potential therapeutic targets, deepening our understanding of their mechanisms in RA development and treatment. The enrichment analysis shows a strong immune functional correlation, consistent with the autoimmune nature of RA.

The current study has certain limitations that warrant consideration. Population stratification and potential sample overlap, inherent in all MR analyses, may introduce bias. Nevertheless, the sensitivity analysis, indicated by the large *F* statistics, suggests that the impact of bias is likely minimal. Moreover, focusing on individuals of European ancestry in the MR analysis may introduce potential bias and restrict the applicability of findings to broader populations. Future research involving diverse population groups will enhance the comprehensiveness of our understanding. Enrichment analysis, while valuable, has inherent limitations as it relies on predefined gene sets or pathways that might not capture the full spectrum of biological mechanisms or interactions. The absence of significant enrichment does not necessarily imply the absence of biological relevance.

The most notable limitation of our genetic analysis is the small effect size (OR ≈ 1.05). Although statistically significant, the biological and clinical relevance of this finding is likely limited. An OR of this magnitude indicates that genetic predisposition to RA explains only a minute fraction of the risk for IDA in the population. It should be interpreted as RA being one of many minor risk factors within a complex etiological landscape, rather than a primary clinical driver. This underscores that the strong clinical association is almost certainly mediated by nongenetic mechanisms. Furthermore, the observed effect size was less pronounced compared to earlier findings in cohort studies, which likely capture the effects of these nongenetic mechanisms. Further exploration, based on summary data from larger GWAS, is warranted for a more precise estimate. In conclusion, utilizing MR analysis on extensive GWAS summary data, we highlight RA as a potential risk factor for IDA in the European ancestry population. Exploring the molecular mechanisms underlying the interaction between peripheral and central immunity may advance our comprehension of the risk of RA on IDA and reveal potential therapeutic targets.

## Acknowledgments

We express gratitude to all contributors for data collection and the authors for their valuable contributions. Special thanks to the participants and investigators of the Database of Genetic Associations from GWAS Summary Datasets Collaborators for their essential involvement in this study.

## Author contributions

**Conceptualization:** Sijie Wang, Yue Lu.

**Data curation:** Sijie Wang, Yue Lu.

**Formal analysis:** Chunlong Yang.

**Methodology:** Sijie Wang, Qiuhua Chen.

**Software:** Chunlong Yang.

**Supervision:** Qingjun Pan.

**Validation:** Qiuhua Chen.

**Writing – original draft:** Sijie Wang, Yue Lu.

**Writing – review & editing:** Qingjun Pan.
